# Acute and subacute intraocular pressure and macular microvascular structure changes after intravitreal ranibizumab injection in eyes with branch retinal vein occlusion

**DOI:** 10.1186/s12886-023-02889-2

**Published:** 2023-04-18

**Authors:** Xiaoyu Li, Qin Chen, Xiaobing Yu

**Affiliations:** 1 Department of Ophthalmology, Beijing Hospital, National Center of Gerontology, Institute of Geriatric Medicine, Chinese Academy of Medical Sciences, Beijing, China; 2 Department of Ophthalmology, West China Hospital of Sichuan University, Chengdu, China

**Keywords:** Intravitreal ranibizumab injection, Intraocular pressure, Macular microvascular structure, Branch retinal vein occlusion

## Abstract

**Purpose:**

To investigate early changes in the intraocular pressure (IOP) and macular microvascular structure in eyes with branch retinal vein occlusion (BRVO) treated with intravitreal Ranibizumab injection.

**Methods:**

This study enrolled 30 patients (one eye per patient) who received intravitreal injections (IVI) of ranibizumab for macular edema secondary to BRVO. IOP were measured before, 30 min (min) and 1 month following IVI. Changes in macular microvascular structure were examined via assessment of foveal avascular zone (FAZ) parameters, vascular density (VD) of superficial vascular complex (SVC), and deep vascular complex (DVC) in whole macula, central fovea and parafovea area which were measured automatically by optical coherence tomography angiography (OCTA) on the same time as IOP examinations. Paired t test and Wilcoxon test were used to compare pre- and post-injection values. The correlation between IOP and OCTA findings was assessed.

**Results:**

IOP Measurements at 30 min post-IVI (17.91 ± 3.36 mmHg) increased significantly from baseline (15.07 ± 2.58 mmHg, p < 0.001), then became similar with baseline after 1 month (15.00 ± 3.16 mmHg, p = 0.925). 30 min past the injection, the parameters of VD of the SCP significantly decreased in comparison to baseline, then became similar with baseline after one month, while there were no significant changes in other OCTA parameters, including parameters of VD of the DCP and the FAZ. At 1 month after IVI, in comparison to baseline, no significant changes were observed in all of the OCTA parameters (P > 0.05). There were no significant correlations between IOP and OCTA findings no matter 30 min or 1 month post-IVI (P > 0.05).

**Conclusions:**

Transient IOP elevation and decreased superficial macular capillary perfusion density were detected 30 min post-IVI, however, no potential continual macular microvascular damage was suspected.

## Introduction

Branch retinal vein occlusion (BRVO) is a relatively common retinal vascular disorder in elderly patients that can lead to visual impairments [1]. Macular edema (ME) secondary to BRVO is considered the main cause of visual impairment [2]. At present, intravitreal anti-vascular endothelial growth factor (VEGF) therapy, such as ranibizumab, is the standard of care of ME associated with BRVO [3]. The usefulness and convenience of IVI of anti-VEGF has been widely recognized [4–7]. However, concerns about the effect of the IVI of anti-VEGF on intraocular pressure (IOP) have arisen in the last few years. It is already known that there is a transient elevation of IOP immediately after any intravitreal injection, which normalizes over minutes to hours [8–12]. Until now, few studies were reported about the acute and subacute post-IVI changes of macular microvascular structure [13]. Whether the acute and subacute post-IVI IOP changes are associated with macular microvascular structure changes is still controversial [14–16]. In this study, we aimed to quantitatively investigate the acute and subacute changes in the IOP and macular microvascular structure in a cohort of patients undergoing intravitreal ranibizumab injection for ME following BRVO, to better understand retinal perfusion changes associated with IVI and also the correlation with the post-IVI IOP changes.

## Materials and methods

### Subjects

This prospective observation study was performed from September, 2016 to October, 2017. The research protocols were approved by the Ethics Committee of Beijing Hospital, carried out in accordance with the tenets of the Declaration of Helsinki. Written informed consent was obtained from each participant. Thirty eyes of 30 patients (one eye per patient) who were ultimately diagnosed with ME due to BRVO were enrolled.

All patients underwent an ophthalmological examination with best-corrected visual acuity (BCVA), IOP measurements, slit lamp examination, fluorescein fundus angiography (FFA), dilated fundoscopic examinations and OCTA. Additionally, vital signs, such as systolic and diastolic blood pressures were also acquired. IOP, OCTA, and blood pressure, including systolic blood pressure (SBP)and diastolic blood pressure (DBP) were acquired at baseline, 30 min, and 1-month post-injection.

Patients were excluded if they met the following criteria: (1) hemi-CRVO or CRVO; (2) diabetic maculopathy and/or retinopathy; (3) any other BCVA compromising ocular disease; (4) any prior intravitreal anti-VEGF or corticosteroid injections; (5) any prior retinal laser photocoagulation; (6) IOP higher than 21 mmHg; (7) history of vitrectomy; (8) history of myocardial infarction or stroke within three months; and (9) other major systemic disorders. The inclusion and exclusion criteria were similar to Song’s study [17].

### Treatments

Each patient received intravitreal ranibizumab injection at a dose of 0.5 mg/ 0.05 ml. After instilling topical 0.4% oxybuprocaine chloride eye drops for topical anesthesia, the eye was irrigated with 5% povidone-iodine and opened using a lid retractor, and the drug was injected through the pars plana 3.5 mm posterior to the limbus in the inferotemporal quadrant in pseudophakic eyes and 4.0 mm posterior to the limbus in phakic eyes using a 30 G needle. The treatment protocol was similar to Song’s study [17].

### IOP

IOP was measured with Goldmann applanation tonometry(Haag-Streit AT 900) at least twice in each eye at each visit. The average of the measurements wastaken. The baseline IOP was measured at the visit before the injection. We also examined the IOP 30 min and 1 month after the injection.

### Optical coherence tomography angiography (OCTA)

**Fig. 1:**
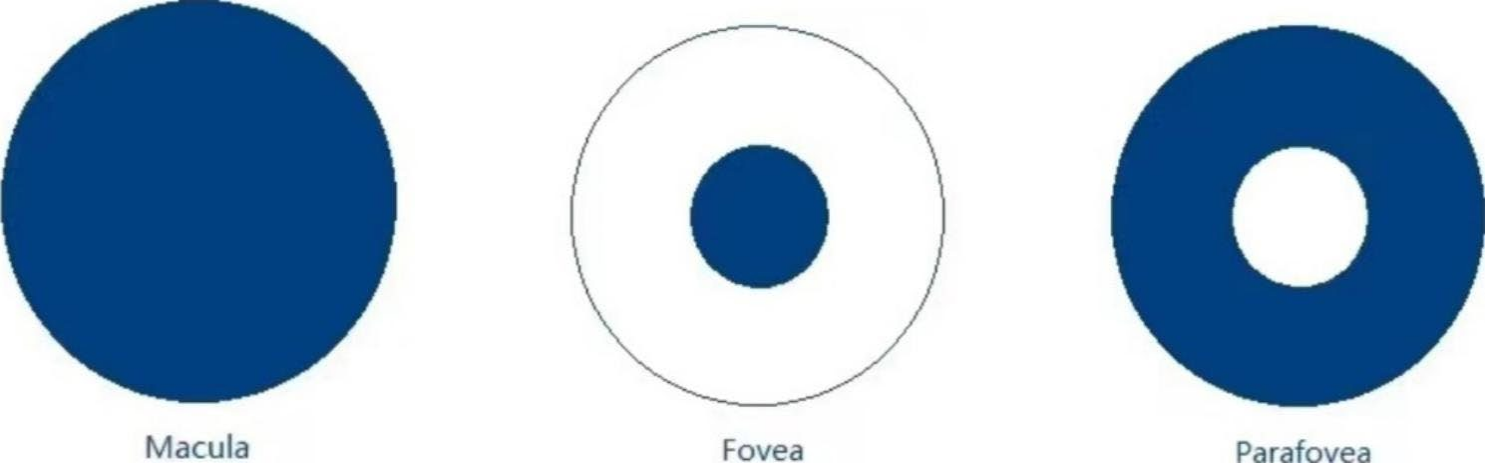
Description of the measured aspects of the 3 × 3 mm macular scans

All patients had macular OCT-A performed before and 30 min, and 1-month post-IVI treatment by spectral-domain OCT (RTVue XR Avanti, Optovue, Inc., Fremont, CA, USA). We only chose OCTA images with an image quality score of 6 or more. For each patient, 3 × 3 mm scans centered on the fovea were acquired. Macular scans were segmented into superficial and deep OCT-A layers. The OCT system’s Angio Analytics software (version 2017.1.0.151, Optovue, Inc.) performs automatic segmentation of vessel layers. Based on the default setting of the OCTA system, the SCP of the retina included blood vessels from the internal limiting membrane (ILM) to − 10 μm below the inner plexiform layer (IPL). DCP of the retina included blood vessels from − 10 μm below the IPL to 10 μm below the outer plexiform layer (OPL). The SCP, DCP as described as the whole macula, the fovea and parafovea which were measured automatically by OCTA built-in software. The fovea was defined as the area within the central 1-mm ring of the Early Treatment Diabetic retinopathy Study (ETDRS) grid. Parafovea was considered as the area between the central 1- and the 3-mm ring of the ETDRS grid. (Fig. 1) FAZ was defined as a capillary-free area in the central macular region on traditional FFA analysis of the retina. FAZ measurements include the following parameters: the FAZ area; the FAZ perimeter (PERIM); the acircularity index (AI), which is defined as the ratio of the perimeter of the FAZ and the perimeter of a circle with equal area, AI = PERIM/equal area standard circumference; and the FD300, which refers to the blood vessel density within a 300 μm wide ring around the FAZ. The FAZ area, PERIM, AI, and FD-300 were used to evaluate the hemodynamics of the FAZ [17]. We recorded the VD of the whole macula, fovea, parafovea of the SCP and DCP, FAZ area, perimeter (PERIM), FD-300 and AI and also their relationships with IOP were observed.

### Statistical analysis

Continuous variables with normal distribution were presented as mean ± standard deviation and with nonnormal distribution were presented as median (interquartile range). Paired t-test and Wilcoxon paired signed rank test were used to compare changes pre- and post-IVI of ranibizumab in BRVO eyes. Normality of errors (residuals) was checked while modeling (histograms and P–P plots). Pearson correlation analyses were adopted to study the correlations among IOP and OCTA findings 30 min and 1 month post-injection. Statistical analyses were performed with the IBM SPSS Statistics for Windows, Version 28.0 (IBM Corp., Armonk, NY, USA), and statistical significance was established at two-tailed P < 0.05.

## Results

30 eyes of 30 patients over age 18 (Mean 57.5 years, Std. Deviation 7.61) were studied. The mean pre-injection IOP was 15.07 ± 2.58 mmHg. The mean IOP at 30 min past the IVI was 17.91 ± 3.36 mmHg with the statistically significant increase of 2.85 mmHg compared with the baseline (p < 0.001). The IOP measurements 1 month post-IVI was 15.00 ± 3.16 mmHg, which decreased significantly compared with 30 min past the injection (mean difference 2.91, p = 0.001), thus became similar with the baseline (mean difference 0.07, p = 0.925). No significant changes were found among the SBP and ABP measurements before, 30 min and 1 month after the IVI(p > 0.05). (Tables 1 and 2).

**Table 1:** Characteristics of the observed values before, 30 min and 1 month after injection

Variables	Pre-injection	30 min Post-injection	1month Post-injection
IOP (mm Hg)	15.07 ± 2.58	17.91 ± 3.36	15.00 ± 3.16
SBP (mm Hg)	130.93 ± 10.71	133.07 ± 12.72	131.13 ± 15.39
DBP (mm Hg)	76.27 ± 7.54	78.53 ± 6.52	77.40 ± 9.69
macular VD in SVC (%)	41.02 ± 3.86	37.93 ± 4.53	42.33 ± 4.50
foveal VD in SVC (%)	17.42 ± 4.35	14.68 ± 5.17	16.19 ± 5.45
parafoveal VD in SVC (%)	42.61 ± 4.27	39.93 ± 4.83	43.49 ± 4.99
macular VD in DVC (%)	42.37 ± 5.61	41.72 ± 5.28	41.34 ± 6.29
foveal VD in DVC (%)	26.91 ± 6.69	24.22 ± 9.22	23.52 ± 6.49
parafoveal VD in SVC (%)	43.97 ± 5.62	44.13 ± 5.04	42.78 ± 6.20
FAZ area(mm2 )	0.329(0.10)	0.35(0.14)	0.37(0.15)
FAZ perimeter(mm)	2.50(0.59)	2.58(0.64)	2.62(0.79)
FAZ AI	1.22(0.1)	1.19(0.18)	1.19(0.21)
FD-300-VD(%)	44.46 ± 5.14	43.89 ± 4.26	44.74 ± 4.17

All values are presented as mean ± SD or median (IQR). IOP: intraocular pressure; SBP: systolic blood pressure; DBP: diastolic blood pressure; SVC: superficial vascular complex; DVC: deep vascular complex; VD: vascular density; FAZ: foveal avascular zone; AI: acircularity index; FD-300-VD: vascular density within a 300 μm wide ring around the foveal avascular zone; SD: standard deviation; IQR: interquartile range

30 min past the injection, statistically significant decreases were found in VD of SVC, as we could see the decreases of VD of SVC in macula (mean difference 3.10, p < 0.001), in fovea (mean difference 2.74, p = 0.006), in parafovea (mean difference 2.68, p = 0.002). 1 month later, the parameters we observed in VD of SVC increased significantly compared with 30 min past the injection, with macula (mean difference 4.41, p < 0.001), fovea (mean difference 1.52, p = 0.007), parafovea (mean difference 3.56, p = 0.009), thus all the superficial parameters became similar with the baseline(p > 0.05). (Tables 1 and 2).

**Table 2:** Differences in the observed values at 30 min and 1 month from baseline

		Time	difference between time 95% CI or Z value	P value
	30 min	0	2.85(1.714, 3.979)	< 0.001
IOP (mm Hg)	1mon	0	-0.067(-1.568, 1.435)	0.925
	1mon	30 min	-2.913(-4.453, -1.374)	0.001
	30 min	0	2.133(-4.205, 8.471)	0.482
SBP (mm Hg)	1mon	0	0.200(-7.782, 8.182)	0.958
	1mon	30 min	-1.933(-9.024,5.157)	0.568
	30 min	0	2.267(-1.567, 6.100)	0.225
DBP (mm Hg)	1mon	0	1.133(-4.463, 6.730)	0.671
	1mon	30 min	-1.133(-6.382, 4.116)	0.650
	30 min	0	-3.098(-4.624, -1.572)	< 0.001*
macular VD in SVC (%)	1mon	0	1.307(-0.843, 3.458)	0.213
	1mon	30 min	4.405(2.081, 6.730)	< 0.001*
	30 min	0	-2.739(-4.547, -0.930)	0.006*
foveal VD in SVC (%)	1mon	0	-1.218(-4.141, 1.705)	0.387
	1mon	30 min	1.521(-2.001, 5.043)	0.007*
	30 min	0	-2.675(-4.206, -1.144)	0.002*
parafoveal VD in SVC (%)	1mon	0	3.593(-1.109, 2.871)	0.309
	1mon	30 min	3.556(1.046, 6.066)	0.009*
	30 min	0	-0.654(-3.335, 2.027)	0.609
macular VD in DVC (%)	1mon	0	-1.036(-4.478, 2.406)	0.529
	1mon	30 min	-0.382(-4.080, 3.316)	0.828
	30 min	0	-2.695(-5.660, 0.269)	0.071
foveal VD in DVC (%)	1mon	0	-3.393(-6.978, 0.192)	0.062
	1mon	30 min	-0.697(-4.607, 3.212)	0.708
	30 min	0	0.165(-2.530, 2.859)	0.898
Parafoveal VD in DVC (%)	1mon	0	-1.191(-4.183, 1.802)	0.408
	1mon	30 min	-1.355(-4.705, 1.994)	0.400
	30 min	0	-1.789	0.074
FAZ area (mm2 )	1mon	0	-1.477	0.140
	1mon	30 min	-1.022	0.307
	30 min	0	-1.193	0.233
FAZ perimeter(mm)	1mon	0	-0.909	0.363
	1mon	30 min	-0.909	0.363
	30 min	0	-0.74	0.459
FAZ AI	1mon	0	-0.094	0.925
	1mon	30 min	-0.683	0.494
	30 min	0	-0.571(-3.461, 2.320)	0.678
FD-300-VD (%)	1mon	0	-0.280(-2.779, 3.339)	0.847
	1mon	30 min	0.851(-1.443, 3.144)	0.440

IOP: intraocular pressure; SBP: systolic blood pressure; DBP: diastolic blood pressure; SVC: superficial vascular complex; DVC: deep vascular complex; VD: vascular density; FAZ: foveal avascular zone; AI: acircularity index; FD-300-VD: vascular density within a 300 μm wide ring around the foveal avascular zone

*P < 0.05

The VD of DVC had no significant changes. No Changes were seen in the VD of DVC in the area of macula, fovea or parafovea no matter 30 min or 1 month after the injection. (p > 0.05) (Tables 1 and 2).

All the FAZ parameters including FAZ area, FAZ perimeter, FAZ AI and FD-300 had no significant changes no matter 30 min or 1 month after the injection. (p > 0.05) (Tables 1 and 2).

Unfortunately, IOP increase had no significant correlation with the decrease in the parameters of VD of SVC, including macula, fovea and parafovea.

## Discussion

IOP elevations are likely to occur in post-IVI cases [8–12]. However, the impact of these transient IOP elevations on retinal physiology is poorly understood. Some authors have shown that IOP spikes and changes in OCTA parameters can be observed post-IV, however, the results are still controversial [14–16]. In this study, we analyzed the acute and subacute alterations in IOP and macular microvascular changes in OCTA parameters in patients with BRVO post-IVI.

In agreement with previous reports, our study shows a transient IOP elevation 30 min after ranibizumab injection. We found the mean pre-injection IOP was 15.07 ± 2.58 mmHg; the mean IOP at 30 min past the IVI was 17.91 ± 3.36 with the significant increase of 2.85 mmHg. The result was similar with many previous studies which have been conducted to assess short-term IOP changes after intravitreal injection [9, 18, 19]. The pathogenesis of short-term increases in IOP after anti-VEGF IVIs is still controversial, but has been presumably due to the transiently increased intraocular fluid volume after injection, thus increases the pressure of the intraocular contents to the eyeball. [9]

The IOP measurements 1 month after the IVI were 15.00 ± 3.16 mmHg, thus became similar with the baseline. Previous studies supported our result. Gado and Macky [20] reported a maximum IOP of 21 mmHg in treated patients over a 3 to 6-month follow-up; Güler et al. [21] reported a mean IOP of 13.2 ± 4.4 mmHg at 1-month follow-up.

In summary, intravitreal injection of anti-VEGF agents generally resulted in acute post-injection elevations of IOP, and in the majority of patients, returned to normal within a short period of time.

Meanwhile, we found that 30 min past the injection, statistically significant decreases were found in VD of SVC, as we could see the decreases of VD of SVC in macula (mean difference 3.10, p < 0.001), in fovea (mean difference 2.74, p = 0.006), in parafovea (mean difference 2.68, p = 0.002), while no changes were seen in the VD of DVC. The result is similar with Barash et al. [15] Recently, Takusagawa et al. [22] found that glaucoma affected the SVC to a much greater degree than DVC. It is not surprising that the SVC VD was greatly reduced in glaucomatous eyes, as it supplies the nerve fiber layer (NFL), the ganglion cell layer (GCL) and part of the inner plexiform layer (IPL), [23–25] which are the anatomic layers most affected by glaucoma [26, 27]. The DCP was minimally affected by glaucoma, as it supplies the middle retinal layers that do not include the retinal ganglion cells.

These changes match with the acute perfusion effects of intravitreal injections observed in our study, where the VD of SVC decreased significantly compared with the VD of DVC, which had no changes. It is possible that we are seeing the ischemic effects of increased IOP. This lends credence to the idea that intravitreal injections may stress the same structures that get damaged in glaucomatous eyes. Since glaucoma damages retinal ganglion cells (RGC) and approximate 1/3 of RGCs resides in the macula, so macular perfusion is theoretically a good place to detect glaucoma and assess disease severity [28, 29].

We also found that no matter superficial or deep retinal capillary perfusion density measured 1 month after IVI of ranibizumab remain unchanged. The result is similar with Falavarajni et al. [30], suggesting that changes like those measured in our study are short term effects more likely to be IOP related than medication related. This study also agrees with other reports that overall FAZ parameters does not change significantly following acute IOP elevation due to intravitreal injections [16].

In the current study, unfortunately, IOP increase had no significant correlation with the decrease in the parameters of VD of SVC, including macula, fovea and parafovea. In our study, the IOP measurements were taken approximately 30 min after injection, whereas the OCT-A imaging followed approximately 1 min thereafter [8–10, 18, 19, 31]. In a study in our department, we measured the IOP of 292 patients 10 min, 30 min after injection and found similar results. The mean IOP was 23.8mmHg at 10 min, and 20.5mmHg at 30 min [32]. In our series, we did not check IOP and OCT-A at 1 min, 5 min or 10-min post-IVI. According to the literature review, we suspect quite possibly, if the OCT-A images and IOP measurements had been taken closer to the time of injection, they may have shown even greater changes in perfusion density and IOP, then maybe we can find the better correlation. As a result, angiographic changes likely correlate to elevated intraocular pressures already in the process of equilibration.

In conclusion, this study of 30 eyes shows that intravitreal injections induce acute changes in IOP and retinal angiographic perfusion density. The superficial layers of the macula are more affected than deep layers by these changes, while after one month, no changes in quantitative OCTA parameters, including, FAZ and VD were observed, thus no potential continual macular microvascular damage was suspected.

The main limitations of this study included the small sample size, the diagnosis with inclusion of only the patients affected by BRVO, the absence of measurements immediately post-IVI. Thus, further randomized controlled studies with a longer follow-up and a larger sample size are highly warranted.

## Data Availability

The datasets used and/or analyzed during the current study are available from the corresponding author upon reasonable request.
